# ATARI trial: ATR inhibitor in combination with olaparib in gynecological cancers with ARID1A loss or no loss (ENGOT/GYN1/NCRI)

**DOI:** 10.1136/ijgc-2021-002973

**Published:** 2021-09-13

**Authors:** Susana Banerjee, James Stewart, Nuria Porta, Christy Toms, Alexandra Leary, Stephanie Lheureux, Saira Khalique, Jeremy Tai, Ayoma Attygalle, Katherine Vroobel, Christopher J Lord, Rachael Natrajan, Judith Bliss

**Affiliations:** 1 Gynaecology Unit, Royal Marsden NHS Foundation Trust, London, UK; 2 Division of Clinical Studies, The Institute of Cancer Research, London, UK; 3 The CRUK Gene Function Laboratory and Breast Cancer Now Toby Robins Breast Cancer Research Centre, The Institute of Cancer Research and The Royal Marsden NHS Foundation Trust, London, UK; 4 Clinical Trials and Statistics Unit, The Institute of Cancer Research, London, UK; 5 Oncology Department, Institut Gustave-Roussy, Villejuif, Île-de-France, France; 6 Division of Medical Oncology and Hematology, University Health Network, Princess Margaret Cancer Centre, Toronto, Ontario, Canada

**Keywords:** ovarian cancer, endometrial neoplasms, cervical cancer

## Abstract

**Background:**

ARID1A (AT-rich interactive domain containing protein 1A) loss-of-function mutations have been reported in gynecological cancers, including rarer subtypes such as clear cell carcinoma. Preclinical studies indicate that *ARID1A* mutant cancers display sensitivity to ATR inhibition while tumors without *ARID1A* mutations may be sensitive to Ataxia telangiectasia and Rad3 related (ATR) inhibitors in combination with poly-ADP ribose polymerase (PARP) inhibitors.

**Primary Objective:**

To determine whether the ATR inhibitor, ceralasertib, has clinical activity as a single agent and in combination with the PARP inhibitor, olaparib, in patients with ARID1A ‘loss’ and ‘no loss’ clear cell carcinomas and other relapsed gynecological cancers.

**Study Hypothesis:**

ARID1A deficient clear cell carcinoma of the ovary or endometrium is sensitive to ATR inhibition, while the combination of ATR and PARP inhibition has activity in other gynecological tumors, irrespective of ARID1A status.

**Trial Design:**

ATARI (ENGOT/GYN1/NCRI) is a multicenter, international, proof-of-concept, phase II, parallel cohort trial assessing ceralasertib activity as a single agent and in combination with olaparib in ARID1A stratified gynecological cancers. Patients with relapsed ovarian/endometrial clear cell carcinoma with ARID1A loss will receive ceralasertib monotherapy (cohort 1A). Relapsed ovarian/endometrial clear cell carcinoma patients with no ARID1A loss (cohort 2) or patients with other histological subtypes (endometrioid, carcinosarcoma, cervical) (cohort 3) will receive combination therapy (olaparib/ceralasertib). Treatment will continue until disease progression.

**Major Inclusion/Exclusion Criteria:**

Patients with histologically confirmed recurrent clear cell (ovarian, endometrial, or endometriosis related), endometrioid (ovarian, endometrial, or endometriosis related), cervical (adenocarcinomas or squamous), or carcinosarcomas (ovarian or endometrial) are eligible. Patients progressing after ≥1 prior platinum with evidence of measurable (RECIST v1.1) radiological disease progression since last systemic anticancer therapy and prior to trial entry are eligible. Previous ATR or PARP inhibitor treatment is not permissible.

**Primary Endpoint:**

Best overall objective response rate (RECIST v1.1).

**Sample Size:**

A minimum of 40 and a maximum of 116.

**Estimated Dates for Completing Accrual and Presenting Results:**

Accrual is anticipated to be complete by the second quarter of 2022, with reporting of results by the fourth quarter of 2022. Overall accrual targets and reporting timelines are dependent on individual cohort progression to stage 2.

**Trial Registration Number:**

NCT0405269.

## Introduction

Cervical, endometrial, and ovarian cancers represent the fourth, sixth, and eighth most commonly diagnosed malignancies among women globally.^1^ Despite high initial response rates to treatment with a combination of surgery and chemotherapy, many patients relapse. The majority of patients that relapse will receive multiple lines of therapy; despite these, diminishing treatment free intervals are common, as is the development of resistant disease, indicating a great unmet need among these patients.

Ovarian, endometrial, and cervical carcinomas have several clinicopathologically distinct histological subtypes, with patients diagnosed with recurrent/metastatic clear cell carcinoma, carcinosarcoma, or cervical cancer displaying the worst outcomes. Recurrent/metastatic ovarian clear cell carcinoma and endometrial clear cell carcinomas are associated with a poor prognosis and resistance to standard cytotoxic chemotherapy.^2^

To date, a number of therapeutic strategies have been trialed in an attempt to improve the outcomes for women diagnosed with ovarian clear cell carcinoma. The NiCCC (ENGOT-0V36) clinical trial, in which patients with clear cell carcinoma were randomized to receive nintedanib, an oral vascular endothelial growth factor, platelet derived growth factor receptor, and fibroblast growth factor receptor inhibitor or standard of care chemotherapy, failed to demonstrate a statistically significant progression free survival or overall survival benefit.^3^ Another therapeutic avenue of interest is immune checkpoint blockade. Phase II studies of programmed cell death protein 1 and programmed death ligand 1 inhibitors have noted durable responses in patients with clear cell histology.^4 5^ The programmed death ligand 1 inhibitor pembrolizumab (Keytruda, MSD) in ovarian clear cell carcinoma is currently being evaluated (NCT03425565).

Loss of function mutations in the tumor suppressor gene *ARID1A* (AT-rich interactive domain containing protein 1A) leading to a loss of protein expression are a frequent observation in both ovarian and endometrial carcinomas of clear cell and endometroid histology (Table 1). ARID1A along with its paralog ARID1B are components of the cBAF complex.^6^ Loss of ARID1A has been shown to result in aberrant cell cycle control.^7^ ARID1A is recruited to double strand DNA breaks via its interaction with ataxia telangiectasia and Rad3 related (ATR). Recruitment of ARID1A to double strand DNA breaks facilitates efficient processing of DNA ends, producing single stranded DNA which becomes coated in replication protein A, thereby sustaining the DNA damage signal and promoting cycle arrest.^8^ A number of novel therapeutic strategies are currently being trialed for ARID1A defective cancers, including poly-ADP ribose polymerase (PARP) inhibitors (NCT03682289), phosphatidylinositol-3-kinase inhibitors (NCT03842228), and bromodomain containing protein 4 (NCT03297424) inhibitors, alongside ATR inhibitors (NCT03682289).

**Table 1:** ARID1A mutation and expression in gynecological cancers

	ARID1A gene mutation	ARID1A loss of expression
Ovarian clear cell carcinoma (%)	35–75 (52)	15–75 (45)
Ovarian endometroid carcinoma (%)	30–63 (47)	31–55 (45)
Endometrial clear cell carcinoma (%)	17	20–26 (21)
Endometrial endometroid carcinoma (%)	40–55 (46)	19–34 (26)
Cervical adenocarcinoma (%)	17	9–31 (12)
Cervical squamous cell carcinoma (%)	7	7–16 (12)
Endometrial carcinosarcoma	20	–
Ovarian carcinosarcoma	80	–

Values are range (median) or median per cent.

ATR is a serine–threonine kinase that plays an essential role in the DNA damage response, detecting single stranded DNA, a common intermediate formed when replication forks stall.^9^ Williamson et al previously demonstrated that small molecule inhibitors of ATR selectively kill tumor cells with defects in the ARID1A. This novel synthetic lethal effect was evident in ovarian clear cell carcinoma cell lines with naturally occurring endogenous *ARID1A* mutations, tumor cell line xenografts, and tumor cell line xenografts derived from *ARID1A* mutant endometrial cancer.^10^ Mechanistically, it has been shown that the topoisomerase enzyme, TOP2A, is localized to DNA in an ARID1A dependent manner and in the absence of ARID1A function, complex chromosomal structures which form during DNA replication which are normally resolved by TOP2A, fail to be processed prior to mitosis.^11^ ATR inhibition in ARID1A defective cells thus activates DNA damage pathways, increases complex chromosomal structures such as anaphase bridges, large scale genomic rearrangements and ultimately causes cell death.^10^

The *BRCA*ness phenotype is not a feature of ovarian clear cell carcinoma; tumors of this subtype do not frequently carry mutations in homologous recombination associated genes, are resistant to platinum based chemotherapy, and lack the genomic scars characteristic of homologous recombination (HR) deficiency. Nevertheless, as previously discussed, ARID1A deficient cancers, such as ovarian clear cell carcinoma, display evidence of an impaired DNA damage response and sensitivity to ATR inhibitors.^10^ In vitro, silencing of ATR has been shown to enhance the cytotoxic effect of PARP inhibitors.^12^ In part, this synergistic interaction has been attributed to the abrogation of ATR mediated cell cycle checkpoints in response to the DNA damaging effects of PARP inhibitors.^13^ Furthermore, ATR inhibition has been shown to overcome PARP inhibitor resistance in *BRCA1* and *BRCA2* mutant cancer cells through both impeding BRCA1 independent loading of *RAD51* at DNA double strand breaks and restoring degradation of stalled replication forks.^14^

The ATARI clinical trial (ATr inhibitor in combination with olaparib in gynecological cancers with ARId1A loss or no loss, NCT04065269) is the first clinical trial which aims to test the hypothesis that ARID1A defective ovarian and endometrial clear cell carcinomas are more sensitive to ATR inhibition. To date, trials aiming to exploit the specific vulnerabilities of ARID1A defective tumors have stratified patients according to their mutation status. However, in ATARI, based on the work of Khalique et al, loss of protein expression, as determined by immunohistochemistry, will be used to stratify patients, followed by retrospective mutation analysis.^15^ Building on the preclinical data which demonstrates synergy between ATR and poly-ADP ribose polymerase inhibition, the combination will be tested in patients with evidence of ARID1A expression (ie, assumed to be wild-type for *ARID1A*).

## Methods

### Trial Design

**Figure 1:**
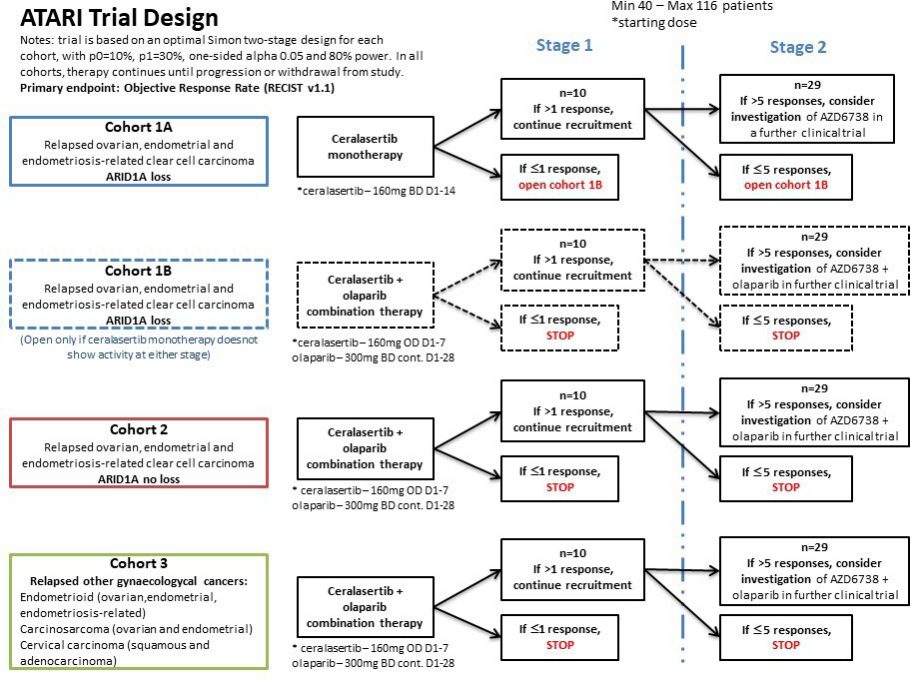
ATARI trial design.

The ATARI clinical trial is an international, academic sponsored phase II clinical trial in which patients with ovarian and endometrial clear cell carcinoma, along with other rare gynecological tumors, are treated with the ATR inhibitor, ceralasertib (AstraZeneca), with or without the PARP inhibitor, olaparib (Lynparza, AstraZeneca) (Figure 1).

The intention is to recruit patients from 18 centers in total (up to six per country) in the UK, France, and Canada. ATARI is sponsored by the Institute of Cancer Research, with central coordination led by the ICR Clinical Trials and Statistics Unit (ICR-CTSU). International participation is coordinated via ARGACY-GINECO (France) and Princess Margaret Hospital Consortium (PMHC) in Canada.

All patients have histological confirmation of clear cell carcinoma by central review following registration and prior to trial entry. Assessment of ARID1A status occurs centrally via immunohistochemistry using archival or fresh tissue biopsies obtained at registration.^15^ Tumors sequenced outside of ATARI, with confirmed ARID1A loss of function mutation, may be eligible for trial entry without repeat immunohistochemistry assessment if testing is performed in an appropriate laboratory (GCLP or CLIA accredited).

Cohort 1A patients are those with clear cell carcinomas (ovarian, endometrial, or endometriosis-related) and demonstrable ARID1A loss. Cohort 1A patients receive ceralasertib monotherapy (160 mg tablets twice daily on days 1–14 in a 28 day cycle). If no activity is observed in this cohort, cohort 1B will open, with the same patient population receiving ceralasertib plus olaparib in combination (160 mg ceralasertib tablets once daily on days 1–7 and 300 mg olaparib tablets twice daily continuously in a 28 day cycle). Patients with clear cell carcinomas (ovarian, endometrial, or endometriosis related) with no ARID1A loss enter cohort 2 and patients with other relapsed gynecological subtypes enter cohort 3, irrespective of ARID1A status. Both cohort 2 and cohort 3 patients receive combination therapy (160 mg ceralasertib tablets once daily on days 1–7 and 300 mg olaparib tablets twice daily continuously in a 28 day cycle).

Each cohort will recruit patients independently, with 10 patients per cohort included in the formal interim analysis at the end of stage 1. If >1 responses are observed, an additional 19 patients will be recruited into the cohort in stage 2. Cohorts 1A/1B, 2, and 3 will be evaluated separately at stage 1 and Stage 2.

In all cohorts, trial treatment continues until disease progression, unacceptable toxicity, withdrawal of consent, or investigator decision that continued treatment is not in the best interest of the patient. Patients are assessed at D1 of each cycle (safety bloods, toxicity assessment) with additional visits at C1D7, C1D15, and C2D15. Tumor imaging and response assessment occurs every 8 weeks.

The trial is funded by AstraZeneca, with additional support provided by the Lady Garden Foundation, and is endorsed by Cancer Research UK.

### Participants

Eligible patients are those with histologically confirmed progressive or recurrent gynecological carcinomas of the following histological sub-types:

- Clear cell (ovarian, endometrial, or endometriosis related)
- Endometrioid (ovarian, endometrial, or endometriosis related)
- Cervical (adenocarcinomas or squamous)
- Carcinosarcomas (ovarian or endometrial)

Patients who have progressed after ≥1 prior platinum containing regimens are eligible, with evidence of measurable (RECIST v1.1) radiological disease progression since the last systemic anticancer therapy and prior to trial entry. Patients previously receiving ATR or PARP inhibitor treatment are excluded.

### Trial Objectives and Endpoints

#### Primary Objective

The primary objective of ATARI is to determine whether ceralasertib has clinical activity, as measured by RECIST 1.1 objective response rate, as a single agent and in combination with olaparib in patients with ARID1A deficient (loss) and no loss relapsed gynecological cancers.

#### Primary Endpoint

The primary endpoint is best overall objective response rate (complete or partial response), as defined by RECIST 1.1. All objective responses reported locally will be centrally reviewed to confirm response.

#### Secondary Objectives

Secondary objectives will evaluate disease control rate (RECIST v1.1) and duration of disease control, progression free survival, time to progression, proportion of patients free of progression at 6 months, and overall survival. Safety and tolerability of ceralasertib as monotherapy and in combination with olaparib in ARID1A loss and no loss relapsed gynecological cancers will be assessed.

#### Exploratory Objectives

Exploratory analyses will assess best percentage change in the sum of target lesions between baseline and while on treatment, and percentage change over time. GCIG (CA125) objective response rate in CA125 evaluable ovarian cancer patients will also be assessed and the correlation between potential tumor and/or circulating biomarkers of clinical activity with ceralasertib monotherapy and the ceralasertib/olaparib combination in gynecological cancers and objective response rate, disease control rate, and progression free survival will be investigated.

#### Sample Size

The total number of patients entered into the trial will be a minimum of 40 and a maximum of 116. Each of the parallel cohorts has been designed following an optimal Simon two stage design to early discard whether ceralasertib monotherapy or in combination with olaparib shows <10% antitumor activity (p=0.01, highest response rate observed in clear cell carcinosarcoma), and powered to show activity over 30% (p1=0.3, clinically meaningful to explore further). Assuming one sided 5% alpha and 80% power, 10 patients will be recruited into each cohort at stage 1. If none or one response is observed, recruitment into the cohort will stop and action will be taken according to Figure 1. If more than one response is observed, 19 additional patients will be recruited in the corresponding cohort into stage 2. If more than five responses are observed overall for the specific cohort at the end of stage 2, it will be considered that the treatment given in that cohort has shown antitumor activity of at least 30% and warrants further investigation. If five or less responses are observed at the end of stage 2, action is taken according to Figure 1.

#### Interim Analysis

The trial is monitored by an independent data monitoring and steering committee who meet every 6 months to oversee the safety of trial participants, monitor data produced by the trial, and oversee progress of the trial towards its interim and overall objectives.

The formal interim analysis will be conducted at the end of stage 1 for each of the stratified cohorts and assessed according to the Simon two stage design, as described above. Stage 1 analysis will be triggered once the target number of evaluable patients has been achieved and after a minimum follow-up of 16 weeks for the last patient entered into the cohort (unless progression status is known sooner).

The independent data monitoring and steering committee will monitor recruitment rate regularly and advise whether halting or pausing recruitment is needed following accrual of 10 patients in each cohort in preparation for interim analysis. If recruitment into one cohort is slow, seamless recruitment into stage 2 would be preferred. Over recruitment into stage 2 will be closely monitored; patients who become unevaluable will be replaced.

### Statistical Methods

Primary analysis of the primary endpoint and key secondary endpoints for each cohort will be triggered once the target number of evaluable patients have had a minimum follow-up of 24 weeks (unless progression status can be ascertained earlier). Analysis will be presented by stratified cohort.

Best overall response and disease control rates will be summarized by number of cases and proportions, reported with exact 95% confidence intervals (CI). For time to event endpoints, Kaplan–Meier graphs and median survival time with 95% CI will be presented.

### Translational Approach

The translational component of ATARI aims to generate data that could help develop how ATR inhibitors are used in the context of clear cell carcinomas and other gynecological cancers. Sequencing of DNA extracted from archival tissue and optional pretreatment and post-progression biopsies where available, will be performed to investigate genetic determinants of ATR inhibitor response and resistance. Plasma samples will be collected from all patients for the duration of study enrollment to evaluate whether biomarkers of ATR inhibitor response can be detected in circulating tumor DNA. Two targeted sequencing panels will be employed to capture known genetic determinants of both ATR inhibitor and PARP inhibitor resistance in circulating tumor DNA.

## Discussion

ATARI is the first clinical trial aiming to determine if the preclinical data showing a synthetic lethal interaction between the tumor suppressor gene *ARID1A* and *ATR* translates into improved outcomes for patients specifically with ovarian and endometrial clear cell carcinoma. The utility of ARID1A protein expression together with mutation status will help determine those patients with functional loss of ARID1A and thus will act as a framework for future clinical trials. The inclusion of the olaparib/ceralasertib combination therapy arm, based on preclinical hypotheses, will investigate whether the two classes of DDR inhibitors have the potential to provide clinical activity worth further exploration. The cohort of non-clear cell, rare gynecological cancers provides the opportunity to assess the combination of ATR and PARP inhibition in a further cohort of patients with limited treatment options. Results from the translational work within this trial will aid both the validation of known biomarkers and identify novel biomarkers of ATR inhibitor response and resistance. The results of the ATARI trial will provide the first indication of whether there is clinical activity of ATR inhibitors in clear cell carcinomas and other rare gynecological cancers according to ARID1A status. These results will help shape future trials and have the potential to change the standard of care in the future for women with rare gynecological cancers.

## Data Availability

There are no data in this work.
